# Mechanistic Clues Provided by Concurrent Changes in the Expression of Genes Encoding the M_1_ Muscarinic Receptor, β-Catenin Signaling Proteins, and Downstream Targets in Adenocarcinomas of the Colon

**DOI:** 10.3389/fphys.2022.857563

**Published:** 2022-03-16

**Authors:** Madeline Alizadeh, Alyssa Schledwitz, Kunrong Cheng, Jean-Pierre Raufman

**Affiliations:** ^1^Division of Gastroenterology and Hepatology, Department of Medicine, University of Maryland School of Medicine, Baltimore, MD, United States; ^2^The Institute for Genome Sciences, University of Maryland School of Medicine, Baltimore, MD, United States; ^3^VA Maryland Healthcare System, Baltimore, MD, United States; ^4^Marlene and Stewart Greenebaum Comprehensive Cancer Center, University of Maryland School of Medicine, Baltimore, MD, United States; ^5^Department of Biochemistry and Molecular Biology, University of Maryland School of Medicine, Baltimore, MD, United States

**Keywords:** colon (C26) carcinoma, muscarinic receptor (subtypes), acetylcholine, G protein-coupled receptors, Wnt/β-catenin signaling pathway

## Abstract

Muscarinic receptors (MRs) in the G protein-coupled receptor superfamily are recipients and mediators of parasympathetic neural transmission within the central and enteric nervous systems. MR subtypes, M_1_R–M_5_R, encoded by *CHRM1-CHRM5*, expressed widely throughout the gastrointestinal (GI) tract, modulate a range of critical, highly regulated activities in healthy tissue, including secretion, motility, and cellular renewal. *CHRM3*/M_3_R overexpression in colon cancer is associated with increased cell proliferation, metastasis, and a worse outcome, but little is known about the role of the other four muscarinic receptor subtypes. To address this gap in knowledge, we queried the NCI Genomic Data Commons for publicly available TCGA-COAD samples collected from colon tissue. RNA-seq data were collected and processed for all available primary adenocarcinomas paired with adjacent normal colon. In this unbiased analysis, 78 paired samples were assessed using correlation coefficients and univariate linear regressions; gene ontologies were performed on a subset of correlated genes. We detected a consistent pattern of *CHRM1* downregulation across colorectal adenocarcinomas. *CHRM1* expression levels were positively associated with those for *APC* and *SMAD4*, and negatively associated with *CTNNB1*, the gene for β-catenin, and with coordinate changes in the expression of β-catenin target genes. These findings implicating *CHRM1*/M_1_R as an important deterrent of colon cancer development and progression warrant further exploration.

## Introduction

Despite meaningful advances in colon cancer prevention and treatment in the United States, metastatic disease remains a highly prevalent cause of morbidity and mortality (Siegel et al., 2021). To address this concern, more must be learned about the molecular mechanisms that modulate progression from primary colon cancer to locoregional and distant metastases. An emerging area, and the focus of our current research, is the role muscarinic receptors (MRs) and alterations in post-MR signaling play in cancer progression (Ali et al., 2021; Schledwitz et al., 2021a; Tolaymat et al., 2021). The five MR subtypes, M_1_R–M_5_R, respectively, encoded by *CHRM1*-*CHRM5*, are G protein-coupled receptors comprised of seven transmembrane helix domains with an extracellular acetylcholine (ACh)-binding region, and extracellular allosteric binding regions for a variety of non-ACh ligands (Maeda et al., 2019; Tolaymat et al., 2021). Of these subtypes, M_1_R and M_3_R, expressed in healthy and neoplastic colon epithelial cells, appear to play prominent roles in disease initiation and progression (Harrington et al., 2010; Cheng et al., 2017; Schledwitz et al., 2021b; Tolaymat et al., 2021). M_3_R activation results in pro-proliferative downstream signaling, and its activity is most clearly associated with colon cancer progression. In mice, functional M_3_R deficiency, whether achieved by ablating *Chrm3* or more recently by selective M_3_R antagonism, reduces tumor number and attenuates cancer cell invasion (Raufman et al., 2008, 2011; Hering et al., 2021). In contrast, M_1_R activation may protect against colon cancer, although its actions are understudied relative to M_3_R (Tolaymat et al., 2021). In mouse models of colon cancer, *Chrm1*/M_1_R deficiency does not reduce tumor size or number. Notably, in mice with combined *Chrm1*/M_1_R and *Chrm3*/M_3_R deficiency, M_1_R deficiency outweighs the effects of M_3_R deficiency; that is mice with combined M_1_R and M_3_R deficiency have as many colon tumors as control mice (Cheng et al., 2014). The molecular biology underlying the differential actions of M_1_R and M_3_R in colon cancer remains obscure.

β-catenin signaling is a critical player in colon cancer pathogenesis; over 90% of sporadic human colon cancers exhibit mutations in at least one protein in the β-catenin pathway - ∼10% exhibit activating mutations in β-catenin and ∼80% have inactivating mutations in APC, a facilitator of cytoplasmic β-catenin degradation (Muzny et al., 2012; Cheng et al., 2019). Our previous work supported the presence of functional interactions between MR and β-catenin signaling (Raufman et al., 2011). The *Apc^*Min*/+^* mouse model generally recapitulates the effects of human β-catenin pathway mutations, although tumors predominate in the small intestine rather than colon (Boivin et al., 2003). Compared to *Apc^*Min*/+^* littermate control mice, we observed that *Chrm3*/M_3_R-deficient *Apc^*Min*/+^* mice had ∼70% fewer small intestinal adenomas (Raufman et al., 2011). Moreover, nuclear staining for β-catenin was significantly reduced in adenomas from *Chrm3*/M_3_R-deficient *Apc^*Min*/+^* mice (Raufman et al., 2011). The mechanistic underpinnings of this interaction between MR and β-catenin signaling were unclear.

To shed light on the role of MR gene expression in colon cancer, we explored a subset of publicly available TCGA RNA-seq data for colon adenocarcinoma and matched normal tissue samples. We found *CHRM1* expression was downregulated in adenocarcinomas and *CHRM1* expression levels correlated with changes in the expression of β-catenin signaling pathway genes and their downstream targets. Gene Ontology analysis applied across all genes associated with *CHRM1* suggested the effects of *CHRM1* downregulation in colon cancer are not subtle, supporting a conceptual framework in which *CHRM1* plays a protective role against colon cancer development.

## Materials and Methods

### Animals

#### M_3_R-Deficient *Apc^*Min*/+^* Mice

As described previously (Raufman et al., 2011), male C57BL/6 *Apc^*Min*/+^* mice (Jackson Laboratory, Bar Harbor, ME, United States) were crossed with female *Chrm3^–/–^* mice on a C57BL/6 genetic background (Taconic Farms). Our breeding schemes yielded *Apc^*Min*/+^Chrm3^+/+^*, *Apc^*Min*/+^Chrm3^+/^*^–^, and *Apc^*Min*/+^Chrm3^–/–^* littermates identified by genotyping using polymerase chain reaction amplification of tail DNA. *Chrm3* primers were M3-A3 (5′-aagaccacagtagcagtg-3′), M3-B (5′ctctctacatccatagtccc-3′), and M_3_-NEO9 (5′-tggatgtggaatgtgtgcgagg-3′). To identify *Apc^*Min*/+^* mice, we used common primer MAPC15 (5′-ttccactttggcataagg-3′), WT primer MAPC9 (5′-gccatcccttcacgttag-3′), and Min primer (5′-ttctgagaaagacagaagtta-3′) mixed in a single polymerase chain reaction (Jackson Labs protocol).

All experiments were approved by the Institutional Animal Care and Use Committee of the University of Maryland, Baltimore, and by the Research and Development Committee at the VA Maryland Health Care System.

### RNA-Seq Data Accession and Processing

Data publicly available on the National Cancer Institute (NCI) Genomic Data Commons were downloaded from the TCGA-COAD analysis of tissue samples of colorectal cancer (Grossman et al., 2016). A subset of these data comprised of paired adenocarcinoma and adjacent normal colon samples were selected for further analysis. Fragments per kilobase of transcript per million mapped reads (FPKM) normalized RNA-seq count data and metadata were collected for 78 primary adenocarcinoma samples with an adjacent normal control (Supplementary Figure 1A). Count tables and metadata were pooled into an expression set and the following processing steps performed using the “hpgltools” package in R (Belew and Hughitt, 2018). Expression data were log2 transformed, quantile normalized, and underwent filtering for low count genes (using the default cbcb filter) prior to SVAseq batch correction (Supplementary Figure 1B). Principal component analysis (PCA) was used to assess appropriateness of the pipeline, and clustering based on condition was present following the pipeline described above (Supplementary Figure 1C).

### Differential Expression and Model Analysis

**FIGURE 1:**
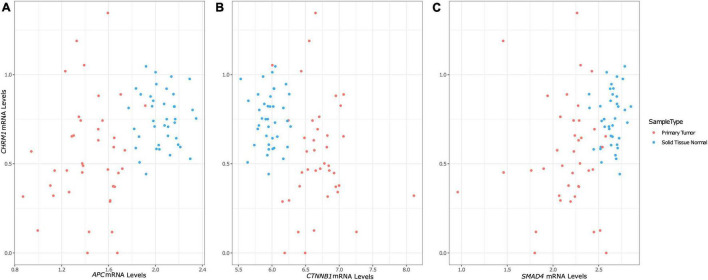
Changes in *CHRM1* mRNA expression levels are associated with changes in the expression levels of *APC, CTNNB1*, and *SMAD4*. We used univariate mixed models with participant ID as a random effect to model processed (low count filtered, quantile normalized, log2 transformed, and batch corrected) *CHRM1* expression data as a function of key genes in colorectal cancer. *CHRM1* expression levels was significantly associated with *APC* **(A)**, *CTNNB1* (encoding β-catenin) **(B)**, and *SMAD4* **(C)** expression levels, which showed differential clustering of values based on cancer status.

Differential expression profiles were generated for the processed adenocarcinoma and matched normal tissue counts using basic log2 fold change calculations, edgeR, and limma-voom analyses for each set of tissues via the “hpgltools” package (McCarthy et al., 2012; Ritchie et al., 2015; Belew and Hughitt, 2018). An adjusted *p*-value using Benjamini Hochberg FDR was applied (Benjamini and Yosef, 1995). Univariate mixed linear models comparing the final processed counts of various genes and *CHRM1*, controlling for participant ID, were generated using the nlme package in R (Pinheiro et al., 2021). *R*^2^-values and residual standard error (RSE) were assessed for all models to ensure generation of a reliable model; residuals were assessed to ensure they were homoscedastic and independent and identically distributed (i.i.d.). The “MuMIn” package was used to generate *R*^2^-values for the models (Bartoń, 2020). For the initial models described in Figure 1, the conditional *R*^2^s ranged from 0.00926 to 0.2330, where a conditional *R*^2^ indicates the variation captured by the model (i.e., *CTNNB1* and *CHRM1* levels, accounting for variation among samples), while the marginal *R*^2^ describes only the variance explained by the fixed effects (i.e., *CTNNB1* and *CHRM1* levels only). Three of the six models produced conditional *R*^2^s suggesting small relationships despite the statistical significance: conditional *R*^2^s of 0.00926, 0.0317, and 0.115 (*Axin1*, *GSK3b*, and *FZD1*, respectively), resulting in no further analysis, and thus in three final models for *APC*, *SMAD4*, and *CTNNB1* (Equations 1–3):


(1) f⁢(C⁢H⁢R⁢M⁢ 1⁢l⁢e⁢v⁢e⁢l⁢s)=βa⁢0+βa⁢1⁢A*⁢P⁢C⁢l⁢e⁢v⁢e⁢l⁢s+εs⁢a⁢m⁢p⁢l⁢e⁢i⁢d

(2) f⁢(C⁢H⁢R⁢M⁢1⁢l⁢e⁢v⁢e⁢l⁢s)=βb⁢0+βb⁢1⁢S*⁢M⁢A⁢D⁢4⁢l⁢e⁢v⁢e⁢l⁢s+εs⁢a⁢m⁢p⁢l⁢e⁢i⁢d

(3) f⁢(C⁢H⁢R⁢M⁢1⁢l⁢e⁢v⁢e⁢l⁢s)=βc⁢0+βc⁢1⁢C*⁢T⁢N⁢N⁢B⁢1⁢l⁢e⁢v⁢e⁢l⁢s+εs⁢a⁢m⁢p⁢l⁢e⁢i⁢d

**FIGURE 2:**
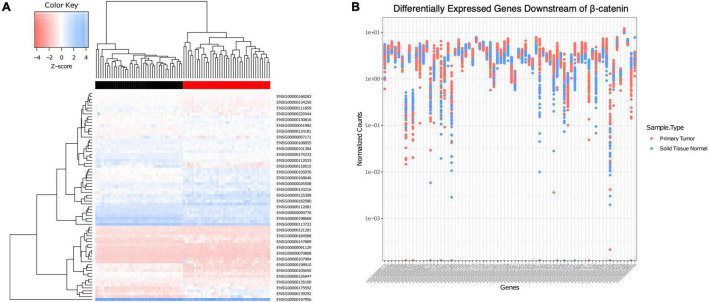
Association of *CHRM1* expression with multiple β-catenin signaling genes predictive of cancer status. **(A)** A heatmap of expression profiles of 72 β-catenin downstream genes demonstrated unsupervised clustering of samples into adenocarcinoma (red) or normal (black) tissue. **(B)** Individual gene expression levels were assessed in each sample, and levels in adenocarcinoma samples were found to be, on average, more varied across all samples.

**FIGURE 3:**
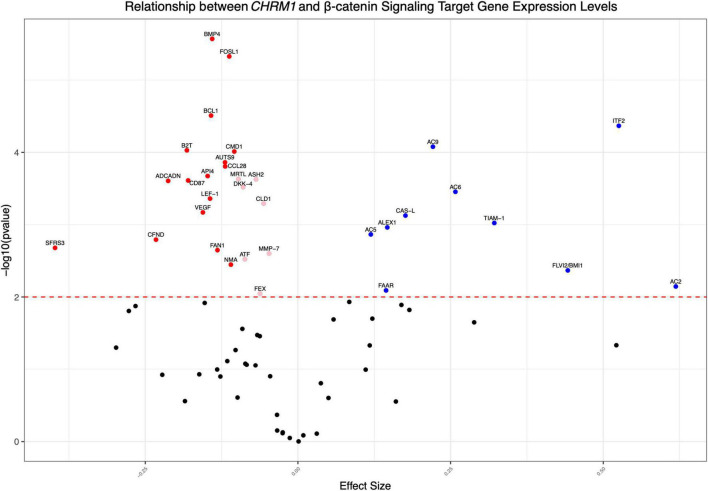
*CHRM1* expression is associated with multiple β-catenin signaling target genes. We modeled *CHRM1* processed expression data as a function of the relevant β-catenin signaling and target genes using mixed (univariate) models, with participant ID as a random effect. β-catenin target genes previously associated with colon cancer and having a significant positive (blue symbols) or negative (red symbols) association with *CHRM1* are shown.

**FIGURE 4:**
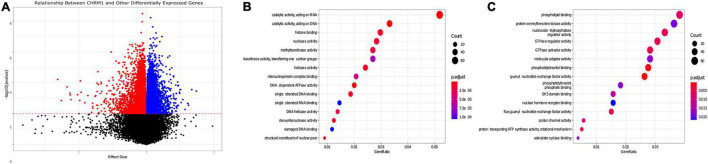
Gene pathways differentially expressed in colon adenocarcinomas associated with changes in *CHRM1* expression levels. **(A)** We used a series of univariate mixed models (participant ID as random effect) to assess the relationship between expression levels of *CHRM1* and other genes in the dataset, with 4,724 genes demonstrating statistically significant (*p*-value ≤ 0.01) negative associations and 4,463 demonstrating significant positive associations with *CHRM1*. **(B)** We detected 3,921 genes that had a model with a surrogate effect magnitude ≥ 0.25 with KEGG data available. Seventy-seven GO terms were upregulated in adenocarcinoma samples—the top 10 are displayed. **(C)** Twenty-five GO terms were downregulated in adenocarcinoma samples.

The RSEs of these ranged from 35.74 to 36.62%, where an RSE describes the typical variance from a model, altogether suggesting Equations 1–3 are predicative but imperfect models (Table 1). “Heatmap.2” was used to generate the heatmap in Figure 2A (Warnes et al., 2020) while the “ggplot2” package was used for all other portions of Figures 1–4 with the exception of Figures 4B,C (Wickham, 2016). The “stats” package in R was used for *t*-tests and to generate Pearson correlation coefficients (R Core Team, 2021). Gene Ontologies were performed using the “gprofiler2” package in R (Kolberg et al., 2020).

**TABLE 1:** Model evaluation of equations described in Table 3.

	RSE	Marginal *R*^2^	Conditional *R*^2^	Pearson correlation coefficient	Residuals iid
*CHRM1*-*APC* model	35.74%	0.1420	0.2330	0.3729 (*p*-value 7.716 E-04)	√
*CHRM1*- *CTNNB1* model	36.72%	0.1223	0.1895	0.3466 (*p*-value 1.878E-03)	√
*CHRM1*- *SMAD4* model	37.02%	0.09763	0.1811	0.3007 (*p*-value 7.468E-03)	√

*Residuals, RSE, and R^2^ of the models described in Table 3 were probed to assess model fit. Residuals were found to be independent, normally distributed, and homoscedastic. Individual Pearson correlation coefficients were all ≥ 0.30, and all models had R^2^ within a reasonable range given the complex interplay and network of genes that is interacting at any given time. Although RSEs were not low, the associative (vs. predictive) intent of our models and the relatively small denominators altogether with the other results suggests the models are reasonable.*

**TABLE 2:** Log2 fold changes in CHRM1–4.

	Basic log2 fold change	Adjusted *p*-value	Limma-voom fold change	Adjusted *p*-value	edgeR fold change	Adjusted *p*-value
*CHRM1*	−0.2067	8.5897E-04	−0.3310	6.044E-05	−0.8446	6.791E-02
*CHRM2*	−0.7694	1.9037E-17	−1.1010	4.462E-21	−2.3380	3.025E-07
*CHRM3*	0.1477	4.2514E-02	0.1046	1.902E-01	0.09757	1.000
*CHRM4*	−0.4018	1.0158E-07	−0.6219	7.594E-11	−1.8890	1.426E-04

*Log2fc was assessed for CHRM1–4 levels in adenocarcinoma vs. solid normal colorectal tissue samples using basic fold changes, limma-voom, and edgeR normalization, respectively, employing the processing scheme illustrated in Supplementary Figure 1B.*

**TABLE 3:** Linear regression results for the expression levels of *CHRM1* and genes commonly mutated in colon cancer.

	Beta coefficient	Standard error	Degrees of freedom	*t*-value	*p*-value
Intercept	0.1752	0.1303	38	1.3444	0.1868
*APC* levels	0.2714	0.7273E-01	38	3.7313	6.2110E-04
Intercept	1.9058	0.3745	38	5.0886	1.0062E-05
*CTNNB1* levels	−0.1985	0.5898E-01	38	−3.3660	1.7553E-03
Intercept	0.70369E-01	0.1978	38	0.3558	0.7239
*SMAD4* levels	0.2415	0.8159E-01	38	2.9597	5.2787E-03

*Univariate linear mixed models with participant id incorporated a random factor generated to model the relationship between CHRM1 and APC, CTNNB1, and SMAD4 levels were statistically significant. FPKM normalized and processed (log2 transformed, low count filtered, quantile normalized, and SVAseq batch corrected) expression levels were used for model generation.*

### Statistical Analysis for Animal Data

Animal data are presented as means ± SE and were analyzed by two-tailed unpaired Student’s *t*-test. *P* < 0.05 were considered statistically significant.

## Results

### *Chrm3* Gene Ablation-Induced Attenuation of β-Catenin Signaling and Intestinal Neoplasia in Mice Identifies Crosstalk Between Muscarinic Receptor and β-Catenin Signaling

Previous evidence supporting a functional interaction between MRs and β-catenin signaling consisted of finding fewer and smaller adenomas in the small intestines of *Apc^*Min*/+^* mice deficient in *Chrm3*/M_3_R (Raufman et al., 2011). Moreover, we observed diminished nuclear staining for β-catenin in these small intestinal adenomas (Raufman et al., 2011). Although *Apc^*Min*/+^* mice are generally considered an animal model of genetic colon cancer, β-catenin signaling is almost equally important in sporadic colon cancer, where approximately 90% of cancers harbor mutations in genes involved in β-catenin signaling. Collectively, these previous findings suggested an important role for MR signaling in the translocation of β-catenin, a key transcription co-factor in colon cancer, to its site of activity in the cell nucleus. Activated β-catenin signaling is known to upregulate the transcription of numerous genes that promote colon neoplasia (Cheng et al., 2019; Chen et al., 2021).

**FIGURE 5:**
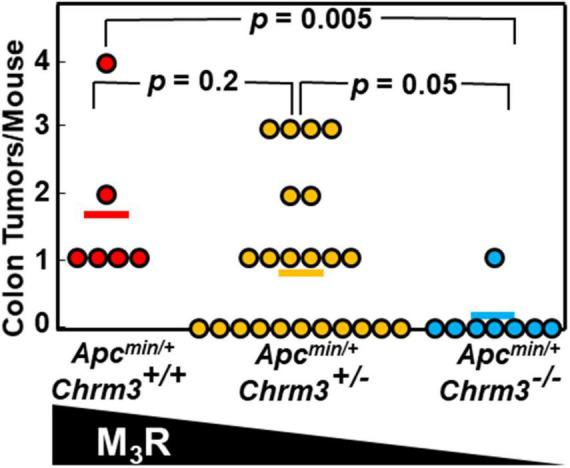
M_3_R deficiency attenuates colon tumor formation in *Apc^*Min*/+^* mice with aberrant β-catenin signaling. In *Apc^*Min*/+^* mice with aberrant β-catenin signaling resulting in exuberant intestinal polyposis, M_3_R deficiency attenuates colon tumor formation. *Apc^*min*/+^* mice with varying M_3_R expression were created as described in Methods. We euthanized mice at 14 weeks of age, harvested colons, and counted and analyzed tumors. Each symbol represents one *Apc^*min*/+^* mouse; bars show means.

To determine if M_3_R deficiency also attenuated neoplasia in *Apc^*Min*/+^* mouse colons, we analyzed colon tumor data recorded during our previous study (Raufman et al., 2011). This previously unpublished analysis revealed that besides impacting the production of small intestinal adenomas, in *Apc^*Min*/+^* mice M_3_R deficiency also robustly attenuated colon neoplasia; we detected colon tumors in all six *Apc^*Min*/+^* mice expressing M_3_R but in only one of eight mice with homozygous M_3_R deficiency (*p* = 0.005; Figure 5). These findings provide strong support for the concept that functional crosstalk between MR and β-catenin signaling robustly impacts the progression of colon neoplasia.

### *CHRM1* Expression Is Attenuated in Human Colorectal Adenocarcinomas

**FIGURE 6:**
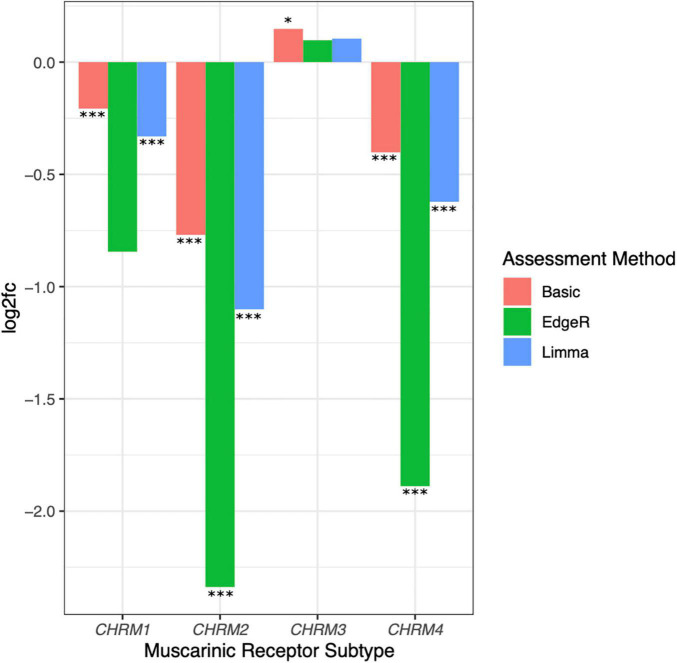
Downregulated expression of *CHRM1*, *CHRM2*, and *CHRM4* and upregulated expression of *CHRM3* mRNA levels in colon cancer. Log2fc was assessed for *CHRM1–4* levels in adenocarcinoma compared to normal colon tissues using basic fold change, limma-voom, and edgeR normalization. Changes in *CHRM2* and *CHRM4* expression were statistically significant (adjusted *p* ≤ 0.05) across all three assessment methods, while changes in *CHRM1* were significant when assessed by basic fold change and limma models; changes in *CHRM3* were only significant using basic log2fc. ***p ≤ 0.001, *p ≤ 0.05.

Comparing muscarinic receptor subtype expression levels in adenocarcinoma vs. normal colon samples, we consistently observed reduced *CHRM1*, *CHRM2*, and *CHRM4* and increased *CHRM3* RNA levels (log2fc normal relative to cancer tissue; Figure 6). Following low count filtration, *CHRM5* expression data were removed from our analysis. Only *CHRM2* and *CHRM4* levels were found to have an adjusted *p* ≤ 0.01 for log2fc across all three analytical methods, while *CHRM1* had an adjusted *p* ≤ 0.01 for all methods except edgeR (Table 2). Since normal tissue samples were derived from full thickness colon tissue samples and M_2_ and M_4_ muscarinic receptor subtypes are predominantly localized in intestinal smooth muscle, we were not surprised to observe reduced *CHRM2* and *CHRM4* expression levels in samples of adenocarcinomas which derive from intestinal epithelial cells; in fact, these findings provided an internal validation of our methodology. Since M_1_R and M_3_R are expressed by both normal and neoplastic intestinal epithelial cells, we considered *CHRM1* and *CHRM3* more disease-relevant candidates for further exploration. Based on these considerations, we explored the relationship between reduced expression of *CHRM1*, increased expression of *CHRM3*, and the expression levels of genes commonly mutated in colorectal cancer.

### *CHRM1* Expression Levels Are Associated With *APC*, *SMAD4*, and Inversely Associated With *CTNNB1* Expression Levels

We first explored the relationship between *CHRM1* levels and *APC*, *KRAS*, *TP53*, and *SMAD4* RNA levels by generating a series of univariate linear mixed models with participant ID as a random factor. The relationship describing *CHRM1* expression values as a function of *KRAS* changes was not statistically significant. Whereas the model of *CHRM1* expression as a function of *TP53* technically showed a significant association, the scatterplot revealed a bimodal distribution of cancer samples along the axis for TP53, suggesting the relationship between *CHRM1* and *TP53* expression is inconsistent in colorectal cancer. Thus, even if a relationship exists, it appears to be independent of cancer status.

In contrast, the model using *APC* expression levels yielded a statistically significant relationship, as did the model using *SMAD4* (*p* ≤ 0.01) (Table 3, Equations 1 and 2, Figures 1A,C), prompting us to investigate the relationship between *CHRM1* levels and other genes relevant to β-catenin signaling, particularly since SMAD4 activates this pathway (Hussein et al., 2003; Romero et al., 2008; Freeman et al., 2012; Du et al., 2020). We detected a statistically significant linear relationship between *CHRM1* and *Axin1*, *CTNNB1* (gene encoding β-catenin; Figure 1B), *FZD1* (gene encoding the Wnt receptor), and *GSK3b* RNA levels (Table 3) using the stringent *p*-value of effect size (beta coefficient) but did not detect statistically significant relationships between *CHRM1* and *WNT*, *GSK3a*, *CK1α*, *DVL1*, *LRP5*, or *LRP6*. A combination of *R*^2^-values, RSE, and assessment of residuals as well as simple Pearson correlation used to assess the models, identified three genes of interest—*APC, SMAD4*, and *CTNNB1* (Equations 1–3).

Performing the same analysis using *CHRM3*, we detected statistically significant linear relationships between increasing *LRP5* and *Axin1* RNA levels, decreasing *GSK3b* RNA levels, and upregulated *CHRM3* expression. However, none of the models were i.i.d. with conditional *R*^2^s ≥ 0.15 and correlation coefficients of ≥ 0.30. As a result of this analysis, moving forward we focused on gene associations with *CHRM1* expression levels.

### Downstream Genes in the β-Catenin Signaling Pathway Predict Cancer Status

To clarify the relationship between *CHRM1* and components of the β-catenin pathway and gauge the potential for functional MR-induced effects on β-catenin signaling, a subset of 72 genes previously identified to be downstream of β-catenin signaling and curated in our dataset were analyzed in normal colon vs. primary colon adenocarcinoma tissues (Herbst et al., 2014). Statistically significant fold changes (adjusted *p* ≤ 0.01) were observed for 58 genes; 23 were downregulated and 35 were upregulated in cancer (Supplementary Table 1). We generated a heatmap of expression levels for those β-catenin-related genes which demonstrated clustering by cancer status (normal vs. adenocarcinoma tissue) using unsupervised clustering, confirming this gene set predicted cancer status (Figure 2A). To examine the potential variation across each individual gene, we generated a plot of the normalized counts for each sample and each gene (Figure 2B). Whereas the variation in expression of some genes was greater in normal compared to cancer samples, most showed a wider range of variation in expression in the cancer samples, suggesting some genes were upregulated and playing a role in a subset of the cancers, but there is variability across samples. We used the paired Student’s *t*-test to analyze these findings, comparing the ranges of normalized values; this revealed a mean increase in variation of 0.7348 in adenocarcinoma (95% CI = 0.4672–1.0023).

### Association of *CHRM1* Expression Levels With Those of Genes in the β-Catenin Signaling Pathway

Using a series of univariate regressions, we explored the relationship between expression levels for the subset of the aforementioned 72 genes and *CHRM1*. Statistically significant (adjusted *p*-value ≤ 0.01) increased RNA expression for 10 (*AC2, AC9*, *ITF2, AC6, CASL, ALEX1, TIAM-1, AC5, FAAR*, and *FLVI2/BMI1*) and decreased RNA expression for 16 genes (*BMP4, FOSL1, BCL1, B2T, CMD1, AUTS9, ADCADN, CD87, API4, CCL28, VEGF, LEF-1, FAN1, NMA, CFND*, and *SFRS3*) were associated with increasing *CHRM1* levels, adjusting for participant ID with a regression coefficient of ≥ 0.1. Regression coefficients were used as surrogate effect sizes given the univariate nature of the mixed model which only controlled for participant ID. Expression levels of an additional seven genes (*MRTL, ASH2, DKK-4, CLD1, MMP-7, ATF*, and *FEX*) were more modest decreased in association with increasing *CHRM1* levels (Figure 3 and Supplementary Table 2).

After expanding the univariate regression analysis to all 36,987 genes, we found 9,187 genes were significantly related to *CHRM1*expression levels—of these, 5,776 had a surrogate effect size with a magnitude ≥ 0.25 (Figure 4A and Supplementary Table 3). Several are pseudogenes, or long noncoding (lnc)RNAs. To identify pathways associated with downregulated *CHRM1* expression in colon cancer we performed a Gene Ontology (GO) analysis on the 5,776 gene subset which also had an adjusted *p*-value of ≤ 0.01 and had available KEGG data (3,921 genes). Of 25 GO terms downregulated in cancer, the following were prominent: phospholipid binding, S/T kinase activity, nucleoside triphosphatase regulator activity, GTPase regulator and activator activity, guanyl nucleotide exchange factor activity, Ras guanyl nucleotide exchange factor activity, and PI and PIP binding (Figure 4C). Of 77 GO terms upregulated in cancer, the following were prominent: catalytic activity on DNA/RNA, histone binding, nuclease and methyltransferase activity, DNA/helicase activity, and single stranded DNA and RNA as well as damaged DNA binding (Figure 4B).

## Discussion

Previous studies implicated MRs in the progression and spread of many cancer types, including colon cancer (Shah et al., 2009). To date, no studies attempted to discern the relationship between MR subtype expression and key players in colon cancer. We sought to fill this gap in knowledge using an unbiased approach to explore expression of MR subtypes in publicly available RNA-seq data. We found *CHRM1* levels were downregulated in colon adenocarcinoma, while, as expected, *CHRM3* was upregulated. Further, we identified an imperfect but linear relationship between *CHRM1* and *APC* and *CTNNB1* mRNA levels, supporting a relationship between *CHRM1*/M_1_R and β-catenin signaling in colon cancer. APC plays a key role in the β-catenin destruction complex to ensure that, in the absence of activation by Wnt ligands, cytosolic β-catenin is ubiquitinated and degraded (Parker and Neufeld, 2020). Notably, Ras degradation is also regulated by GSK3β, a member of the β-catenin destruction complex (Lee et al., 2018). As M_1_R activation results in downstream Ras/MAPK activation (Qian et al., 1995), this illuminates a potential node of intersection between M_1_R and β-catenin signaling.

Although M_3_R activation results in similar downstream cascades to those modulated by M_1_R activation, M_3_R expression is upregulated in colorectal adenocarcinoma and promotes cancer progression. The divergent effects of M_1_R vs. M_3_R activation on colon cancer were described previously (Tolaymat et al., 2021) and remain poorly understood. Notably, other differences between the expression and actions of these MR subtypes are reported. For example, M_1_R and M_3_R differ in their propensity to engage in cAMP signaling via non-canonical pathways. Following treatment with a muscarinic agonist, Chinese hamster ovary (CHO) cells expressing only M_1_R maximally accumulate four times as much cAMP as CHO cells expressing only M_3_R, despite similar receptor density, pharmacokinetics, and efficiency of signaling via phospholipase C, inositol triphosphate, and calcium. Investigation of the underlying mechanism suggested post-M_1_R stimulation of cAMP accumulation involves activation of G_s_ (Burford et al., 1995; Burford and Nahorski, 1996). As β-catenin is a target of phosphorylation by PKA, which is activated by cAMP (Katoh and Katoh, 2017), reduced expression of *CHRM1*/M_1_R in colon cancer would attenuate β-catenin signaling mediated by this non-canonical M_1_R/cAMP/PKA/β-catenin axis. This differential impact of M_1_R and M_3_R activation on cAMP signaling adds additional complexity and potential fine tuning to the impact of MR expression and activation on β-catenin signaling.

It is possible that differential localization of MRs within cells comprising the tumor microenvironment also plays a role in discrepant effects; M_3_R is more consistently expressed throughout multiple tissue layers, including circular and longitudinal muscle, myenteric nerve cell bodies, and mucosal epithelial tissue, while M_1_R expression is largely restricted to myenteric and submucosal nerve cells and epithelial cells (Harrington et al., 2010). In support of this, the wide variation in identified expression levels in *CHRM3* in colon cancer, specifically across a range of experimental methods and cell lines, suggests tissue localization may play a role in the observed changes (Felton et al., 2018; Ali et al., 2021). Further, unlike the other muscarinic receptor subtypes, both M_1_R and M_3_R undergo pre-coupling with G_i/o_ G-proteins, that are their non-preferential G proteins. Differential pre-coupling in different tissues could result in variable downstream effects following MR activation (Jakubík et al., 2011).

Our exploration of the relationship between *CHRM1* and genes known to be differentially expressed downstream of β-catenin (Figure 3) was similarly thought-provoking. Despite great variation across expression levels of those genes, particularly in cancer tissue, they remained predictive of cancer status. By exploring the relationship between those genes and *CHRM1* levels, we identified a subset of genes with a statistically significant association. Univariate analyses identifying concurrent changes in gene expression suggested functional interactions. Of particular interest were positive correlations with *ITF2* and *AC2*, which had the highest surrogate effect sizes. *ITF2* is implicated as both a tumor suppressor and proto-oncogene in colon cancer (Davidsen et al., 2018); relevant to the current analysis, in human colon cancer cells and tissue, ITF2 is reported to prevent activation of the β-catenin-TCF4 complex and transcription of β-catenin gene targets (Shin et al., 2014). Decreased levels of *AC2* and the general class of adenylyl cyclases was previously demonstrated in human colon cancer cell lines (Nelson and Holian, 1988; Yi et al., 2018; Fan et al., 2019). These changes in *AC2* expression may also be relevant to the non-canonical M_1_R/cAMP/PKA/β-catenin axis described above.

Additionally, we identified a negative correlation with *SFRS3*, implicated previously in colon cancer (Kuranaga et al., 2018; Zhou et al., 2020). We detected more modest inverse correlations with *BMP4* and *FOSL1* (but with higher conditional *R*^2^-values and high *p*-values); these genes are also implicated in colon cancer cell proliferation and metastasis (Diesch et al., 2014; Yokoyama et al., 2017; Liu et al., 2021). Notably, *MMP7*, has a well-documented role in colon cancer progression and metastasis (Kitamura et al., 2009; Koskensalo et al., 2011; Polistena et al., 2014), as does VEGF via vasculature remodeling in tumor-adjacent tissues (Ellis et al., 2000; Liu et al., 2011; Ahluwalia et al., 2014; Yang et al., 2015). Notably, our group previously observed that M_3_R activation induces *MMP7* expression in human colon cancer cells (Xie et al., 2009). Hence, the inverse relationship between *CHRM1* and *MMP7* expression levels is of particular interest. While we anticipated detecting genes previously implicated in colon cancer, the relative relationship to changes in *CHRM1* expression suggests a common relationship between the concurrent changes observed in *CHRM1* and *APC/CTNNB1*, and these downstream β-catenin target genes.

Lastly, we used the relationship between expression levels of genes with a significant relationship to *CHRM1* expression to identify gene ontology (GO) terms and pathways of importance to cancer progression. Several GPCR regulation- and activation-related GO terms were downregulated, as were several potentially *CHRM1* cascade-related GO terms such as PI and PIP binding and guanyl nucleotide exchange factor activity. Although these genes were specifically stratified based on altered levels correlating with changes in *CHRM1* expression, it remains interesting that changes in *CHRM1* and related genes had sufficient impact to associate with downregulated pathways. While the sheer number of genes with changing expression levels paralleling *CHRM1* was high, the fact that the most concurrently downregulated pathways were *CHRM1*-related while the most concurrently upregulated GO terms were growth- and proliferation-related, implies the strong correlations were unlikely due to chance and reflect the impact of *CHRM1* downregulation in colon cancer. This further validates that *CHRM1*/M_1_R downregulation observed in colon cancer is not only non-random, but very likely involved in important aspects of development and/or progression of disease.

Our findings suggest an important relationship exists between *CHRM1*/M_1_R and β-catenin signaling in colorectal cancer. Nonetheless, we acknowledge important limitations. Our analytical approach cannot identify a specific mechanism (s) that underlies directionality of gene changes nor a specific cause and effect relationship. Moreover, our results do not exclude the possibility that additional players are involved; additional studies are required to elucidate the processes driving these changes. For example, RNA-seq experiments in *Chrm1*/M_1_R knockout mice may elucidate the temporal dynamics and confirm the directionality of such changes in gene and protein expression, thus better delineating the mechanisms whereby these pathways intersect.

Collectively, these patterns of changes in gene expression levels and cancer-related pathways support a conceptual framework wherein *CHRM1*/M_1_R expression contributes to protection against the development and progression of colorectal adenocarcinoma. Further elucidation of these novel mechanistic insights regarding intersection of *CHRM1*/M_1_R and β-catenin signaling, beyond the scope of the current project, warrant additional experimentation.

## Data Availability Statement

The datasets analyzed for this study can be found in the Genomic Data Commons, at https://portal.gdc.cancer.gov/, under the TCGA-COAD project.

## Author Contributions

MA and J-PR conceptualized the project. KC performed the experiments and analyzed the results. MA, AS, and J-PR wrote the initial draft, proofread, edited, contributed additional material, and completed the final draft. MA, AS, KC, and J-PR approved the final manuscript. All authors contributed to the article and approved the submitted version.

## Author Disclaimer

The contents do not represent the views of the U.S. Department of Veterans Affairs or the U.S. Government.

## Conflict of Interest

The authors declare that the research was conducted in the absence of any commercial or financial relationships that could be construed as a potential conflict of interest.

## Publisher’s Note

All claims expressed in this article are solely those of the authors and do not necessarily represent those of their affiliated organizations, or those of the publisher, the editors and the reviewers. Any product that may be evaluated in this article, or claim that may be made by its manufacturer, is not guaranteed or endorsed by the publisher.

## References

[B1] Ahluwalia A. Jones M. K. Matysiak-Budnik T. Tarnawski A. S. (2014). VEGF and colon cancer growth beyond angiogenesis does VEGF directly mediate colon cancer growth via a non-angiogenic mechanism? *Curr. Pharm. Des.* 20 1041–1044. 10.2174/1381612819999131218175905 23755727

[B2] Ali O. Tolaymat M. Hu S. Xie G. Raufman J. P. (2021). Overcoming obstacles to targeting muscarinic receptor signaling in colorectal cancer. *Int. J. Mol. Sci.* 22:716. 10.3390/ijms22020716 33450835 PMC7828259

[B3] Belew A. Hughitt K. (2018). *“hpgltools, A Pile of (hopefully) Useful R Functions.” R package version 2018.03.* Available online at: https://rdrr.io/github/elsayed-lab/hpgltools/man/hpgltools.html

[B4] Bartoń K. (2020). *“MuMIn, Multi-Model Inference.” R Package Version 0.12.2/r18.* Available at online at: https://r-forge.r-project.org/R/?group_id=346 (accessed December 23, 2021).

[B5] Benjamini Y. Yosef H. (1995). Controlling the false discovery rate a practical and powerful approach to multiple testing. *J. R. Stat. Soc. B* 57 289–300.

[B6] Boivin G. P. Washington K. Yang K. Ward J. M. Pretlow T. P. Russell R. (2003). Pathology of mouse models of intestinal cancer consensus report and recommendations. *Gastroenterology* 124 762–777. 10.1053/gast.2003.50094 12612914

[B7] Burford N. T. Nahorski S. R. (1996). Muscarinic m1 receptor-stimulated adenylate cyclase activity in Chinese hamster ovary cells is mediated by Gs alpha and is not a consequence of phosphoinositidase C activation. *Biochem. J.* 315 883–888. 10.1042/bj3150883 8645172 PMC1217289

[B8] Burford N. T. Tobin A. B. Nahorski S. R. (1995). Differential coupling of m1 m2 and m3 muscarinic receptor subtypes to inositol 1,4,5-trisphosphate and adenosine 3’,5’-cyclic monophosphate accumulation in Chinese hamster ovary cells. *J. Pharmacol. Exp. Ther.* 274 134–142. 7616390

[B9] Chen G. T. Tifrea D. F. Murad R. Habowski A. N. Lyou Y. Duong M. R. (2021). Disruption of β-Catenin-Dependent wnt signaling in colon cancer cells remodels the microenvironment to promote tumor invasion. *Mol. Cancer Res*. 10.1158/1541-7786.MCR-21-0349 [Epub ahead of print]. PMC8898281 34799404

[B10] Cheng K. Shang A. C. Drachenberg C. B. Zhan M. Raufman J. P. (2017). Differential expression of M3 muscarinic receptors in progressive colon neoplasia and metastasis. *Oncotarget* 8 21106–21114. 10.18632/oncotarget.15500 28416748 PMC5400569

[B11] Cheng K. Xie G. Khurana S. Heath J. Drachenberg C. B. Timmons J. (2014). Divergent effects of muscarinic receptor subtype gene ablation on murine colon tumorigenesis reveals association of M3R and zinc finger protein 277 expression in colon neoplasia. *Mol. Cancer* 13:77. 10.1186/1476-4598-13-77 24694019 PMC4021221

[B12] Cheng X. Xu X. Chen D. Zhao F. Wang W. (2019). Therapeutic potential of targeting the Wnt/β-catenin signaling pathway in colorectal cancer. *Biomed. Pharmacother.* 110 473–481. 30530050 10.1016/j.biopha.2018.11.082

[B13] Davidsen J. Larsen S. Coskun M. Gögenur I. Dahlgaard K. Bennett E. P. (2018). The VTI1A-TCF4 colon cancer fusion protein is a dominant negative regulator of Wnt signaling and is transcriptionally regulated by intestinal homeodomain factor CDX2. *PLoS One* 13:e0200215. 10.1371/journal.pone.0200215 29975781 PMC6033461

[B14] Diesch J. Sanij E. Gilan O. Love C. Tran H. Fleming N. I. (2014). Widespread FRA1-dependent control of mesenchymal transdifferentiation programs in colorectal cancer cells. *PLoS One* 9:e88950. 10.1371/journal.pone.0088950 24658684 PMC3962334

[B15] Du X. Li Q. Yang L. Liu L. Cao Q. Li Q. (2020). SMAD4 activates Wnt signaling pathway to inhibit granulosa cell apoptosis. *Cell Death Dis.* 11:373. 10.1038/s41419-020-2578-x 32415058 PMC7228950

[B16] Ellis L. M. Takahashi Y. Liu W. Shaheen R. M. (2000). Vascular endothelial growth factor in human colon cancer, biology and therapeutic implications. *Oncologist* 5 (Suppl. 1), 11–15. 10.1634/theoncologist.5-suppl_1-11 10804085

[B17] Fan Y. Mu J. Huang M. Imani S. Wang Y. Lin S. (2019). Epigenetic identification of ADCY4 as a biomarker for breast cancer an integrated analysis of adenylate cyclases. *Epigenomics* 11 1561–1579. 10.2217/epi-2019-0207 31584294

[B18] Felton J. Hu S. Raufman J. P. (2018). Targeting M3 muscarinic receptors for colon cancer therapy. *Curr. Mol. Pharmacol.* 11 184–190. 10.2174/1874467211666180119115828 29357811 PMC6371400

[B19] Freeman T. J. Smith J. J. Chen X. Washington M. K. Roland J. T. Means A. L. (2012). Smad4-mediated signaling inhibits intestinal neoplasia by inhibiting expression of β-catenin. *Gastroenterology* 142 562–571.e2. 10.1053/j.gastro.2011.11.026 22115830 PMC3343368

[B20] Grossman R. L. Heath A. P. Ferretti V. Varmus H. E. Lowy D. R. Kibbe W. A. (2016). Toward a shared vision for cancer genomic data. *N. Engl. J. Med.* 375 1109–1112. 10.1056/NEJMp1607591 27653561 PMC6309165

[B21] Harrington A. M. Peck C. J. Liu L. Burcher E. Hutson J. M. Southwell B. R. (2010). Localization of muscarinic receptors M1R M2R and M3R in the human colon. *Neurogastroenterol. Motil.* 22 999–1008. 10.1111/j.1365-2982.2009.01456.x 20146726

[B22] Herbst A. Jurinovic V. Krebs S. Thieme S. E. Blum H. Göke B. (2014). Comprehensive analysis of β-catenin target genes in colorectal carcinoma cell lines with deregulated Wnt/β-catenin signaling. *BMC Genomics* 15:74. 10.1186/1471-2164-15-74 24467841 PMC3909937

[B23] Hering N. A. Verena L. Rayoung K. Benjamin W. Raoul D. A. Marco A. (2021). Blockage of cholinergic signaling via muscarinic acetylcholine receptor 3 inhibits tumor growth in human colorectal adenocarcinoma. *Cancers* 13:3220. 10.3390/cancers13133220 34203220 PMC8267754

[B24] Hussein S. M. Duff E. K. Sirard C. (2003). Smad4 and beta-catenin co-activators functionally interact with lymphoid-enhancing factor to regulate graded expression of Msx2. *J. Biol. Chem.* 278 48805–48814. 10.1074/jbc.M305472200 14551209

[B25] Jakubík J. Janíčková H. Randáková A. El-Fakahany E. E. Doležal V. (2011). Subtype differences in pre-coupling of muscarinic acetylcholine receptors. *PLoS One* 6:e27732. 10.1371/journal.pone.0027732 22110745 PMC3218020

[B26] Katoh M. Katoh M. (2017). Molecular genetics and targeted therapy of WNT-related human diseases (Review). *Int. J. Mol. Med.* 40 587–606. 10.3892/ijmm.2017.3071 28731148 PMC5547940

[B27] Kitamura T. Biyajima K. Aoki M. Oshima M. Taketo M. M. (2009). Matrix metalloproteinase 7 is required for tumor formation, but dispensable for invasion and fibrosis in SMAD4-deficient intestinal adenocarcinomas. *Lab. Invest.* 89 98–105. 10.1038/labinvest.2008.107 19002110

[B28] Kolberg L. Raudvere U. Kuzmin I. Vilo J. Peterson H. (2020). gprofiler2- an R package for gene list functional enrichment analysis and namespace conversion toolset g:Profiler. *F1000Res.* 9:ELIXIR-709. 10.12688/f1000research.24956.2 33564394 PMC7859841

[B29] Koskensalo S. Louhimo J. Nordling S. Hagström J. Haglund C. (2011). MMP-7 as a prognostic marker in colorectal cancer. *Tumour Biol.* 32 259–264. 10.1007/s13277-010-0080-2 21207220

[B30] Kuranaga Y. Sugito N. Shinohara H. Tsujino T. Taniguchi K. Komura K. (2018). SRSF3 a Splicer of the PKM Gene, regulates cell growth and maintenance of cancer-specific energy metabolism in colon cancer cells. *Int. J. Mol. Sci.* 19:3012. 10.3390/ijms19103012 30279379 PMC6213643

[B31] Lee S. K. Jeong W. J. Cho Y. H. Cha P. H. Yoon J. S. Ro E. J. (2018). β-Catenin-RAS interaction serves as a molecular switch for RAS degradation via GSK3β. *EMBO Rep.* 19:e46060. 10.15252/embr.201846060 30413483 PMC6280641

[B32] Liu W. Xu J. Wang M. Wang Q. Bi Y. Han M. (2011). Tumor-derived vascular endothelial growth factor (VEGF)-a facilitates tumor metastasis through the VEGF-VEGFR1 signaling pathway. *Int. J. Oncol.* 39 1213–1220. 10.3892/ijo.2011.1138 21785819

[B33] Liu Y. Yue M. Li Z. (2021). FOSL1 promotes tumorigenesis in colorectal carcinoma by mediating the FBXL2/Wnt/β-catenin axis via Smurf1. *Pharmacol. Res.* 165:105405. 10.1016/j.phrs.2020.105405 33450386

[B34] Maeda S. Qu Q. Robertson M. J. Skiniotis G. Kobilka B. K. (2019). Structures of the M1 and M2 muscarinic acetylcholine receptor/G-protein complexes. *Science* 364 552–557. 10.1126/science.aaw5188 31073061 PMC7034192

[B35] McCarthy D. J. Chen Y. Smyth G. K. (2012). Differential expression analysis of multifactor, RNA-Seq experiments with respect to biological variation. *Nucleic Acids Res.* 40 4288–4297. 10.1093/nar/gks042 22287627 PMC3378882

[B36] Muzny D. M. Matthew N. B. Kyle C. Huyen H. D. Jennifer A. D. Gerald Fowler C. L. (2012). Comprehensive molecular characterization of human colon and rectal cancer. *Nature* 487 330–337. 10.1038/nature11252 22810696 PMC3401966

[B37] Nelson R. L. Holian O. (1988). Adenylate cyclase activity and cyclic adenosine monophosphate levels in colon cancer lines and dermal fibroblasts and the effects of cholera toxin and epidermal growth factor. *J. Surg. Oncol.* 38 108–112. 10.1002/jso.2930380211 2837611

[B38] Parker T. W. Neufeld K. L. (2020). APC controls Wnt-induced β-catenin destruction complex recruitment in human colonocytes. *Sci. Rep.* 10:2957. 10.1038/s41598-020-59899-z 32076059 PMC7031393

[B39] Pinheiro J. Bates D. DebRoy S. Sarkar D. R Core Team (2021). *_nlme, Linear and Nonlinear Mixed Effects Models_.”R Package Version 3.1-155*, Available online at: https://CRAN.R-project.org/package=nlme (accessed December 23, 2021).

[B40] Polistena A. Cucina A. Dinicola S. Stene C. Cavallaro G. Ciardi A. (2014). MMP7 expression in colorectal tumours of different stages. *In Vivo* 28 105–110. 24425843

[B41] Qian N. X. Russell M. Johnson G. L. (1995). Acetylcholine muscarinic receptor regulation of the Ras/Raf/MAP kinase pathway. *Life Sci.* 56 945–949. 10.1016/0024-3205(95)00032-2 10188797

[B42] R Core Team (2021). *R: A Language and Environment for Statistical Computing.* (Vienna: R Foundation for Statistical Computing).

[B43] Raufman J. P. Samimi R. Shah N. Khurana S. Shant J. Drachenberg C. (2008). Genetic ablation of M3 muscarinic receptors attenuates murine colon epithelial cell proliferation and Neoplasia. *Cancer Res.* 68 3573–3578. 10.1158/0008-5472.CAN-07-6810 18483237 PMC2577901

[B44] Raufman J. P. Shant J. Xie G. Cheng K. Gao X. M. Shiu B. (2011). Muscarinic receptor subtype-3 gene ablation and scopolamine butylbromide treatment attenuate small intestinal neoplasia in Apcmin/+ mice. *Carcinogenesis* 32 1396–1402. 10.1093/carcin/bgr118 21705482 PMC3165126

[B45] Ritchie M. E. Phipson B. Wu D. Hu Y. Law C. W. Shi W. (2015). Limma powers differential expression analyses for RNA-sequencing and microarray studies. *Nucleic Acids Res.* 43:e47. 10.1093/nar/gkv007 25605792 PMC4402510

[B46] Romero D. Iglesias M. Vary C. P. Quintanilla M. (2008). Functional blockade of Smad4 leads to a decrease in beta-catenin levels and signaling activity in human pancreatic carcinoma cells. *Carcinogenesis* 29 1070–1076. 10.1093/carcin/bgn054 18310088

[B47] Schledwitz A. Sundel M. H. Alizadeh M. Hu S. Xie G. Raufman J. P. (2021a). Differential actions of muscarinic receptor subtypes in gastric, pancreatic, and colon cancer. *Int. J. Mol. Sci.* 22:13153. 10.3390/ijms222313153 34884958 PMC8658119

[B48] Schledwitz A. Xie G. Raufman J. P. (2021b). Exploiting unique features of the gut-brain interface to combat gastrointestinal cancer. *J. Clin. Invest.* 131:e143776. 10.1172/JCI143776 33998603 PMC8121521

[B49] Shah N. Khurana S. Cheng K. Raufman J. P. (2009). Muscarinic receptors and ligands in cancer. *Am. J. Physiol. Cell Physiol.* 296 C221–C232. 10.1152/ajpcell.00514.2008 19036940 PMC2643856

[B50] Shin H. W. Choi H. So D. Kim Y. I. Cho K. Chung H. J. (2014). ITF2 prevents activation of the β-catenin-TCF4 complex in colon cancer cells and levels decrease with tumor progression. *Gastroenterology* 147 430–442.e8. 10.1053/j.gastro.2014.04.047 24846398

[B51] Siegel R. L. Miller K. D. Fuchs H. E. Jemal A. (2021). Cancer Statistics, 2021. *CA Cancer J. Clin.* 71 7–33. 33433946 10.3322/caac.21654

[B52] Tolaymat M. Margaret S. H. Madeline A. Guofeng X. Jean-Pierre R. (2021). Potential role for combined subtype-selective targeting of M1 and M3 muscarinic receptors in gastrointestinal and liver diseases. *Front. Pharmacol.* 12:786105. 10.3389/fphar.2021.786105 34803723 PMC8600121

[B53] Warnes G. R. Bolker B. Bonebakker L. Gentleman R. Huber W. Liaw A. Lumley T. Maechler M. (2020). *“gplots, Various R Programming Tools for Plotting Data.* Available at online at: https://github.com/talgalili/gplots (accessed December 23, 2021).

[B54] Wickham H. (2016). *ggplot2, Elegant Graphics for Data Analysis.* Berlin: Springer-Verlag.

[B55] Xie G. Cheng K. Shant J. Raufman J. P. (2009). Acetylcholine-induced activation of M3 muscarinic receptors stimulates robust matrix metalloproteinase gene expression in human colon cancer cells. *Am. J. Physiol. Gastrointest. Liver Physiol.* 296 G755–G763. 10.1152/ajpgi.90519.2008 19221016 PMC2670666

[B56] Yang X. Zhang Y. Hosaka K. Andersson P. Wang J. Tholander F. (2015). VEGF-B promotes cancer metastasis through a VEGF-A-independent mechanism and serves as a marker of poor prognosis for cancer patients. *Proc. Natl. Acad. Sci. U.S.A.* 112 E2900–E2909. 10.1073/pnas.1503500112 25991856 PMC4460438

[B57] Yi H. Wang K. Jin J. F. Jin H. Yang L. Zou Y. (2018). Elevated adenylyl cyclase 9 expression is a potential prognostic biomarker for patients with Colon Cancer. *Med. Sci. Monit.* 24 19–25. 10.12659/msm.906002 29292367 PMC5759510

[B58] Yokoyama Y. Watanabe T. Tamura Y. Hashizume Y. Miyazono K. Ehata S. (2017). Autocrine BMP-4 signaling is a therapeutic target in colorectal cancer. *Cancer Res.* 77 4026–4038. 10.1158/0008-5472.CAN-17-0112 28611046

[B59] Zhou Z. Gong Q. Lin Z. Wang Y. Li M. Wang L. (2020). Emerging Roles of SRSF3 as a therapeutic target for cancer. *Front. Oncol.* 10:577636. 10.3389/fonc.2020.577636 33072610 PMC7544984

