# Supplementary material for: Mechanistic Clues Provided by Concurrent Changes in the Expression of Genes Encoding the M1 Muscarinic Receptor, β-Catenin Signaling Proteins, and Downstream Targets in Adenocarcinomas of the Colon
Source: Front Physiol. 2022 Mar 16;13:857563. doi: 10.3389/fphys.2022.857563 (PMC8966224; doi:10.3389/fphys.2022.857563)
Supplement: Supplementary file 4 [file Presentation_1.PPTX]

## Slide 1
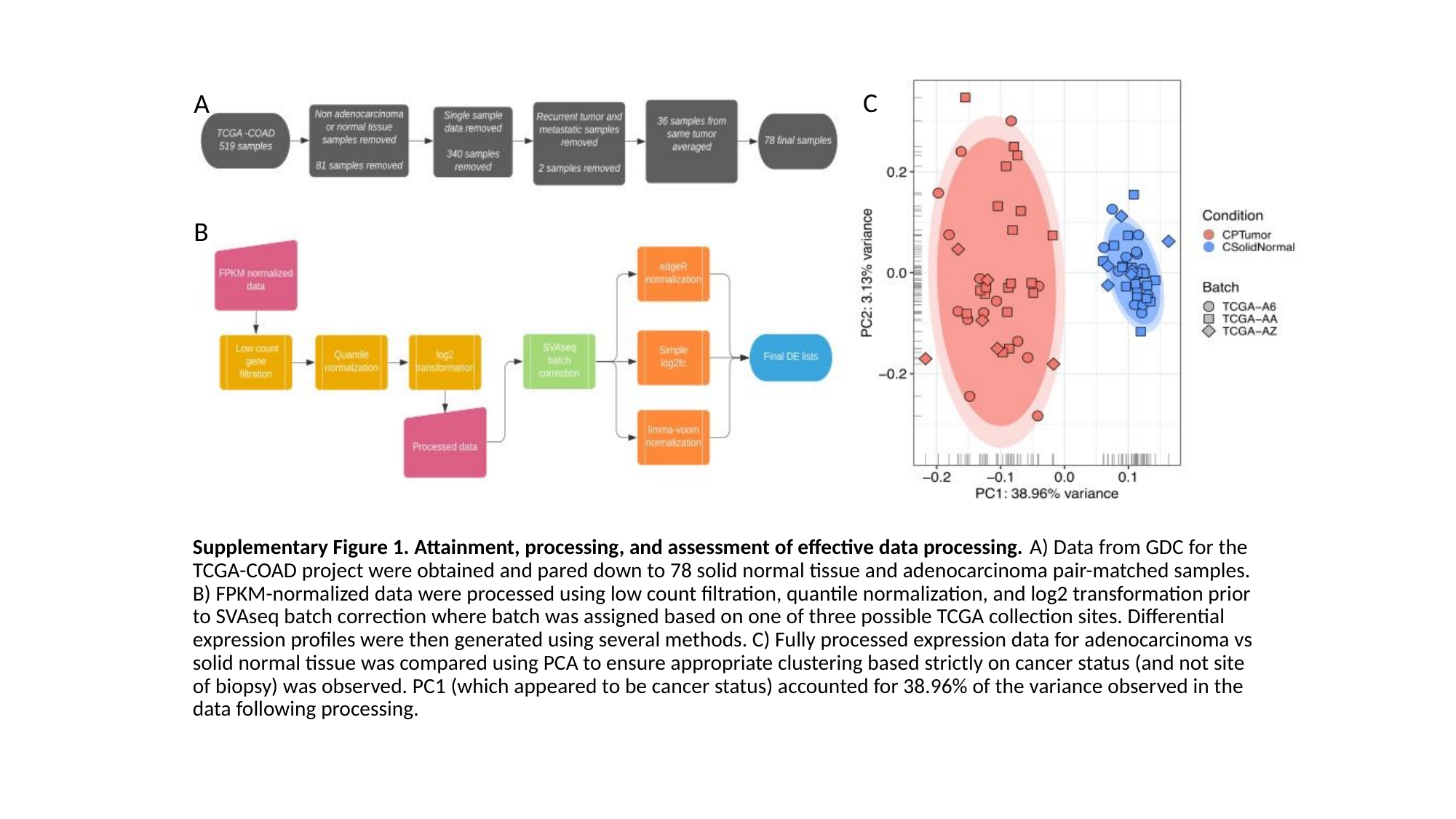

C
A
B
Supplementary Figure 1. Attainment, processing, and assessment of effective data processing. A) Data from GDC for the TCGA-COAD project were obtained and pared down to 78 solid normal tissue and adenocarcinoma pair-matched samples. B) FPKM-normalized data were processed using low count filtration, quantile normalization, and log2 transformation prior to SVAseq batch correction where batch was assigned based on one of three possible TCGA collection sites. Differential expression profiles were then generated using several methods. C) Fully processed expression data for adenocarcinoma vs solid normal tissue was compared using PCA to ensure appropriate clustering based strictly on cancer status (and not site of biopsy) was observed. PC1 (which appeared to be cancer status) accounted for 38.96% of the variance observed in the data following processing.
